# Reproductive Outcomes Following Minimally Invasive Surgery for Deep Endometriosis: A Cohort Study

**DOI:** 10.3390/jcm15145384

**Published:** 2026-07-09

**Authors:** Andrei Manu, Elena Poenaru, Arina-Ilinca Gheorghe, Smaranda Stoleru, Alexandra Irma Gabriela Baușic, Bogdan-Cătălin Coroleucă, Ciprian-Andrei Coroleucă, Cristina-Maria Iacob, Mihaela Arina Banu, Anca-Mihaela Hashemi, Maria-Bianca Nițescu, Oana-Miruna Peiu, Elvira Brătilă

**Affiliations:** 1 Doctoral School, “Carol Davila” University of Medicine and Pharmacy, 020021 Bucharest, Romania; andrei.manu@drd.umfcd.ro (A.M.); cristina-maria.iacob@drd.umfcd.ro (C.-M.I.); mihaela-arina.banu@drd.umfcd.ro (M.A.B.); 2 Clinical Hospital of Obstetrics and Gynecology “Prof. Dr. Panait Sîrbu”, 060251 Bucharest, Romania; alexandra.bausic@umfcd.ro (A.I.G.B.); ccoroleuca@yahoo.com (B.-C.C.); anca-mihaela.hashemi@rez.umfcd.ro (A.-M.H.); maria-bianca.nitescu@rez.umfcd.ro (M.-B.N.); oana-miruna.peiu@rez.umfcd.ro (O.-M.P.); elvirabarbulea@gmail.com (E.B.); 3 Faculty of Medicine, “Carol Davila” University of Medicine and Pharmacy, 050474 Bucharest, Romania; arina-ilinca.gheorghe0721@stud.umfcd.ro (A.-I.G.); smaranda.stoleru@umfcd.ro (S.S.)

**Keywords:** deep endometriosis (DE), colorectal endometriosis, fertility-sparing surgery, segmental resection, Spontaneous pregnancy, nerve-sparing technique, endometriosis fertility index (EFI)

## Abstract

**Objective**: To evaluate reproductive outcomes following fertility-sparing surgery for deep endometriosis (DE), specifically assessing the impact of bowel resection on spontaneous conception rates and the predictive value of the Endometriosis Fertility Index (EFI). **Design**: Retrospective observational cohort study. Setting: High-volume tertiary referral center for endometriosis. Patients: A total of 507 women with histologically confirmed endometriosis and documented infertility or active desire for pregnancy, managed between 2018 and 2025. Patients undergoing hysterectomy were excluded. **Interventions**: Laparoscopic complete excision of endometriotic lesions using a nerve-sparing technique. The surgical strategy for bowel involvement was tailored to nodule characteristics: 194 patients (38.3%) underwent segmental colorectal resection, 38 (7.5%) underwent rectal shaving, and 9 (1.8%) were treated with advanced organ-sparing techniques (lateral rectal resection or extra-mucosal excision—EMEB). **Main Outcome Measures**: Postoperative pregnancy rate (PR), mode of conception (spontaneous vs. ART), and factors influencing fertility. **Results**: The cohort presented with severe disease (mean rASRM stage 3.4) and a high prevalence of primary infertility (79.3%). During the follow-up period, 310 patients achieved pregnancy, resulting in an overall pregnancy rate of 61.1%. Notably, 70.3% of these pregnancies were achieved spontaneously. Radicality did not compromise fertility: the segmental resection group achieved a pregnancy rate of 91.2% (177/194), while patients treated with rectal shaving achieved 100%. The EFI score was identified as a robust predictor of success (mean score 5.5 in pregnant vs. 4.9 in non-pregnant patients, *p* < 0.05). **Conclusions**: Comprehensive nerve-sparing excision of DE, including segmental bowel resection, is associated with high pregnancy rates and a predominant restoration of natural fertility. Surgery should be considered a first-line strategy to reduce dependency on assisted reproductive technologies.

## 1. Introduction

Endometriosis is a complex, estrogen-dependent inflammatory disorder characterized by the presence of endometrial-like tissue outside the uterine cavity, affecting approximately 10% of women of reproductive age and reaching prevalence rates of up to 50% among women undergoing evaluation for infertility [1]. The burden of this disease extends beyond pain symptomatology, profoundly impacting reproductive potential through multifactorial mechanisms that continue to challenge contemporary gynecological practice [1,2].

Deep endometriosis (DE) constitutes the most severe phenotypic manifestation of this disease, defined by lesions penetrating more than 5 mm beneath the peritoneal surface [3]. These invasive lesions frequently involve anatomically critical structures, including the bowel, uterosacral ligaments, rectovaginal septum, and urinary tract, creating profound therapeutic dilemmas regarding the optimal balance between disease eradication and organ preservation [4]. The association between DE and subfertility has been extensively documented, with emerging evidence suggesting that the severity of anatomical involvement correlates with the magnitude of reproductive impairment [5,6,7].

The pathophysiological mechanisms underlying endometriosis-associated infertility are increasingly recognized as multidimensional rather than solely anatomical. At the molecular level, ectopic endometrial tissue exhibits aberrant estrogen and progesterone receptor signaling, resistance to programmed cell death mediated by anti-apoptotic proteins such as Bcl-2, and heightened proliferative capacity reflected by elevated Ki-67 indices [8], alterations that collectively compromise endometrial receptivity and drive disease progression, consistent with the characterization of endometriosis as a systemic disorder with broad reproductive implications [2,8,9].

The diagnostic approach to DE has evolved substantially with advances in imaging technology. High-resolution transvaginal ultrasound (TVUS), when performed by trained operators using systematic evaluation protocols, has demonstrated diagnostic accuracy comparable to magnetic resonance imaging (MRI) for the detection and mapping of deep endometriotic lesions, particularly those involving the rectosigmoid colon [10]. This diagnostic precision enables individualized preoperative planning and comprehensive patient counseling regarding surgical complexity and anticipated morbidity [10,11].

Minimally invasive surgical excision is central to the management of endometriosis-associated infertility, aiming to restore pelvic anatomy and remove inflammatory lesions [3,12]. Cohort studies and systematic reviews suggest comprehensive excision enhances spontaneous conception in selected patients [5,13,14], although optimal management of bowel endometriosis remains debated [4,15]. Colorectal involvement requires balancing radical excision against preservation of bowel function. Conservative techniques (rectal shaving, discoid excision) carry lower perioperative morbidity, whereas segmental resection may be required for large, multifocal, or stenotic nodules [16,17]. When performed with high-volume teams using nerve-sparing techniques, radical approaches do not appear to compromise fertility relative to conservative strategies [16,17,18], and the growing role of ART continues to shape the debate on optimal sequencing in women with bowel involvement [19,20]. Surgery-first proponents argue that restoring pelvic anatomy addresses the underlying pathophysiology while also treating pain, reducing reliance on costly ART [2,21], whereas ART-first advocates emphasize time efficiency, particularly for women with limited reproductive lifespan or compromised ovarian reserve [20,22]. This decision should ultimately be guided by patient-specific factors such as age, ovarian reserve, tubal status, and surgical complexity [19,20,23].

Beyond the immediate achievement of surgical success, the systematic assessment of postoperative fertility potential has emerged as a critical component of patient management. The Endometriosis Fertility Index (EFI), developed and validated by Adamson and Pasta, integrates historical factors (age, duration of infertility), surgical findings (least function score for tubes, fimbriae, and ovaries), and anatomical characteristics (American Society for Reproductive Medicine stage) to generate a predictive score for spontaneous pregnancy following surgical treatment [24,25]. Multiple subsequent validation studies across diverse populations have confirmed the robustness of the EFI as a clinical decision tool, demonstrating its utility in postoperative counseling and in guiding the timing of ART referral [25,26].

The impact of surgical treatment extends beyond fertility restoration to encompass broader dimensions of patient well-being. Minimally invasive excision of DE has been demonstrated to significantly improve multiple domains of quality of life, including pain symptomatology, physical functioning, emotional well-being, and sexual function, as measured by validated assessment instruments [27,28]. These multidimensional benefits underscore the value proposition of surgery as a comprehensive therapeutic intervention rather than solely a fertility-enhancing procedure. The prospective evaluation of quality of life outcomes using standardized tools has become increasingly recognized as an essential component of outcomes research in endometriosis [27,28]. Translational evidence further implicates peritoneal immune dysregulation and altered endometrial gene expression in endometriosis-associated infertility, with surgical excision proposed to favorably modulate this inflammatory milieu and thereby enhance both natural and ART-mediated conception [9,29,30].

The evolving surgical approach to bowel endometriosis reflects a paradigm shift toward “tailored radicality”—the concept that surgical technique should be adapted to nodule characteristics with the dual objectives of achieving complete disease clearance while maximizing organ preservation. Classic techniques such as rectal shaving, while minimizing bowel-related morbidity, may leave residual disease with potential for persistent symptoms or recurrence [15,31]. In contrast, advanced organ-sparing techniques, including lateral rectal resection (for eccentric nodules) and extra-mucosal excision of bowel endometriosis (EMEB, for deep muscular infiltration with intact mucosa), represent refinements aimed at achieving oncologic-style radicality while preserving bowel circumference and luminal integrity [4,15]. Segmental resection, when indicated for extensive disease, achieves definitive excision but carries risks including anastomotic complications and low anterior resection syndrome (LARS) [16,17,32]. The decision-making process regarding surgical technique requires synthesis of multiple factors, including nodule size, degree of stenosis, distance from the anal verge, and surgeon expertise [4,15,31].

Long-term reproductive outcomes following surgical management of DE have been investigated in several longitudinal cohort studies with extended follow-up periods. Data from five-year follow-up analyses suggest that the fertility-enhancing effects of comprehensive surgical excision are durable, with cumulative pregnancy rates continuing to increase over time, particularly in women with preserved ovarian reserve, although deep pelvic disease itself may adversely affect ovarian reserve and oocyte yield [18,33,34]. However, the impact of recurrent disease on fertility outcomes remains an area requiring further investigation, particularly regarding the optimal management of patients experiencing symptomatic recurrence following primary surgery [22,35].

The role of ART in the management of endometriosis-associated infertility has been extensively studied, with particular focus on outcomes following failed surgical treatment or in populations where surgery is deemed inappropriate [22,35,36]. Data from large registry studies indicate that women with endometriosis generally achieve lower per-cycle pregnancy rates with in vitro fertilization (IVF) compared to women with tubal factor infertility, though cumulative live birth rates after multiple cycles can approach acceptable thresholds [22,36]. Interestingly, retrospective analyses suggest that women who undergo complete surgical excision of endometriosis prior to IVF may experience improved ART outcomes compared to those proceeding directly to IVF, possibly reflecting resolution of the inflammatory microenvironment or improved ovarian response [9,30]. This observation has generated renewed interest in the concept of surgery as a “reset” intervention capable of optimizing subsequent ART success [30].

The present study addresses these knowledge gaps by evaluating fertility outcomes in a large, well-characterized cohort of patients treated for DE using contemporary minimally invasive surgical techniques. Our specific objectives are threefold: (1) to quantify the overall pregnancy rate and characterize the relative contributions of spontaneous conception versus ART to successful pregnancies; (2) to assess the comparative impact of different surgical approaches to bowel endometriosis, including segmental resection and advanced organ-sparing techniques, on fertility outcomes; and (3) to validate the predictive utility of the Endometriosis Fertility Index and other clinical variables in this contemporary surgical cohort. By providing comprehensive, real-world data from a high-volume referral center, we aim to inform clinical decision-making and contribute evidence-based guidance for the counseling of women with DE-associated infertility.

## 2. Materials and Methods

### 2.1. Study Design and Settings

This retrospective observational cohort study included women undergoing minimally invasive surgery for deep endometriosis between January 2018 and December 2025 at two tertiary referral hospitals in Bucharest, Romania (Clinical Hospital of Obstetrics and Gynecology ‘Prof. Dr. Panait Sârbu’ and Memorial Hospital). The study protocol was approved by the Institutional Ethics Committee. The research was conducted in strict accordance with the ethical standards laid down in the 1964 Declaration of Helsinki and its later amendments. Informed consent for the use of clinical and medical data for research purposes was obtained from all participants prior to surgery.

### 2.2. Patient Selection

The study enrolled patients with histologically confirmed endometriosis who underwent fertility-sparing surgery within the aforementioned timeframe. A total of 1024 patients were initially screened. To ensure a homogenous cohort focused on reproductive outcomes, strict selection criteria were applied. In total, 507 patients met predefined eligibility criteria and constituted the final analysis cohort. For the purposes of this study, infertility was defined in accordance with the ESHRE criteria as the failure to achieve a clinical pregnancy after 12 or more months of regular, unprotected sexual intercourse [1]. Primary infertility was defined as the absence of any prior clinical pregnancy, while secondary infertility was defined as infertility following at least one previous pregnancy, regardless of outcome. Women who had not yet attempted conception but expressed an active desire for future pregnancy at the time of surgical consultation were classified separately as having a documented desire for conception and were included given the fertility-sparing nature of the intervention and the relevance of surgical decision-making to their reproductive planning. Of the 507 patients included, 464 (91.5%) had a documented diagnosis of primary or secondary infertility, while the remaining 43 (8.5%) had not yet attempted conception but expressed an active desire for future pregnancy. Given the small size of this latter subgroup, fertility outcomes were analyzed for the cohort as a whole rather than stratified separately by infertility status.

Inclusion criteria: Women of reproductive age (18–45 years) with histologically confirmed endometriosis; documented primary or secundary infertility and/or a preoperative or postoperative desire for conception; elective minimally invasive surgery (laparoscopic or robotic) with complete excision of endometriotic lesions; preservation of the uterus and at least one functional ovary; Availability of postoperative fertility follow-up data (minimum 12 months).

Exclusion criteria: Concurrent hysterectomy or bilateral oophorectomy; diagnosis of pelvic malignancy or borderline ovarian tumor; absence of desire for conception; severe male factor infertility (azoospermia) precluding assessment of natural conception potential; follow-up duration insufficient to determine pregnancy outcome.

### 2.3. Surgical Management

All surgical interventions were performed by a single high-volume multidisciplinary team under the direction of the same senior surgeon, operating across both institutions, ensuring consistency in the surgical technique and decision-making process. The approach was strictly minimally invasive, adhering to the principles of complete macroscopic excision of all visible lesions. The surgical strategy was “tailored” to the extent of the disease, with a specific focus on nerve-sparing dissection to preserve the hypogastric and pelvic splanchnic nerves, essential for reproductive function.

When bowel involvement was present, the choice of technique was individualized based on nodule characteristics (size and depth), degree of muscularis involvement and lumen compromise, multifocality, and anatomical location. Rectal shaving was used for superficial lesions without significant muscular invasion; lateral rectal resection was performed for selected infiltrative lesions requiring partial-thickness excision; and extra-mucosal excision (EMEB) was used in selected cases to achieve lesion removal while preserving rectal mucosal integrity without full-thickness resection. Segmental colorectal resection was performed for lesions with substantial muscular infiltration and/or significant luminal compromise, according to standard colorectal surgical principles.

Concurrent surgical gestures, such as ovarian cystectomy (using stripping technique with maximal preservation of ovarian cortex) and chromopertubation to assess tubal patency, were performed when indicated. Postoperative morbidity data, including anastomotic complications, were recorded but are reported separately as they fall outside the primary scope of this fertility-focused analysis. Postoperative morbidity data were systematically recorded for all patients undergoing bowel surgery. Major complications were uncommon: anastomotic bleeding requiring reoperation occurred in 1.2% of the segmental resection subgroup, anastomotic leak in 0.3%, pelvic abscess requiring reintervention in 0.3%, and transient postoperative bladder atony in 0.6%, with no cases of ureteral fistula or stoma formation.

### 2.4. Data Collection and Outcomes

Clinical, imaging, and operative data were retrieved from institutional electronic databases and operative reports. The primary outcome was the postoperative clinical pregnancy rate (PR), defined as the proportion of women achieving at least one clinical pregnancy during follow-up. Clinical pregnancy was defined as ultrasound confirmation of an intrauterine gestational sac (with or without fetal cardiac activity, depending on the available documentation).

Secondary outcomes included: (1) mode of conception, categorized as spontaneous (natural intercourse without medical intervention) or medically assisted; and (2) the association between postoperative Endometriosis Fertility Index (EFI) score and pregnancy achievement. For the purposes of this analysis, medically assisted conception was defined as pregnancy achieved through intrauterine insemination (IUI) or in vitro fertilization/intracytoplasmic sperm injection (IVF/ICSI). These modalities are reported as a composite ART category; the proportion of IUI versus IVF/ICSI pregnancies within this group is detailed in the Results section.

The EFI score was calculated postoperatively for each patient by the operating surgeon according to the original validated methodology [24], integrating historical factors (age, duration of infertility, prior pregnancy) and intraoperative findings (least function score for adnexa bilaterally, rASRM stage). Fertility follow-up was conducted at approximately 6, 12, and 24 months after surgery through scheduled clinical visits; when pregnancy occurred or was managed outside the study centers, pregnancy status and mode of conception were ascertained from available medical documentation (e.g., ultrasound report, obstetric records, or ART clinic documentation) whenever possible.

### 2.5. Statistical Analysis

Statistical analysis was performed using IBM SPSS Statistics for Windows, Version 28.0 (IBM Corp., Armonk, NY, USA). Descriptive statistics were used to characterize the study cohort: continuous variables (Age, AMH, EFI) were expressed as mean ± standard deviation (SD), while categorical variables (Infertility type, Surgical procedure) were presented as frequencies and percentages. To evaluate predictors of fertility success, we compared the “Pregnant” vs. “Non-Pregnant” groups using the independent Student’s *t*-test for continuous variables and the Chi-square test for categorical variables. A *p*-value of <0.05 was considered statistically significant.

## 3. Results

### 3.1. Study Population and Clinical Characteristics

**Figure 1:**
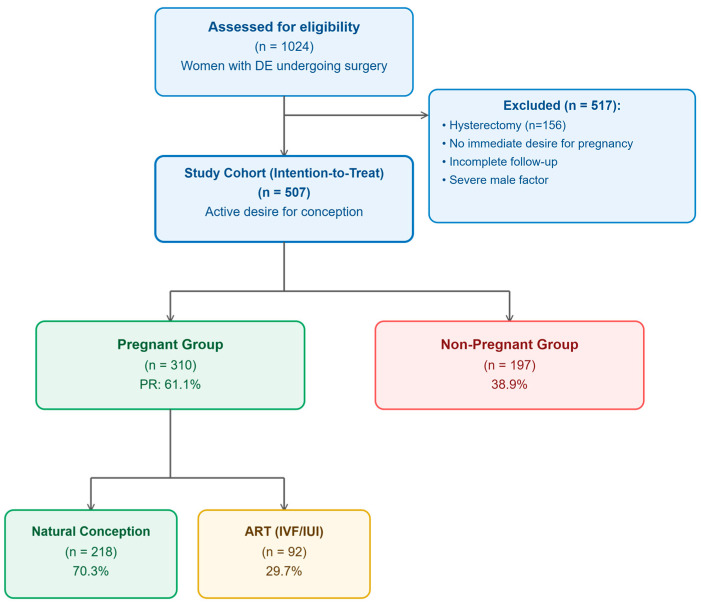
The patient selection process and study flow.

Patient Recruitment and Selection Process: This retrospective cohort study was conducted at Clinical Hospital of Obstetrics and Gynecology “Prof. Dr. Panait Sarbu” and Memorial Hospital, Bucharest, a tertiary referral center specializing in deep endometriosis (DE). From a total initial pool of 1024 patients surgically managed for endometriosis between 2018 and 2025, after applying predefined eligibility criteria, 156 women who underwent hysterectomy were excluded and a further 361 patients were excluded due to lack of documented pregnancy intention and/or insufficient reproductive follow-up to ascertain pregnancy outcome. The final analysis cohort comprised 507 women undergoing fertility-sparing surgery (Figure 1).

The baseline clinical and demographic profile of the cohort is summarized in Table 1. The mean age at the time of surgery was 33.0 ± 5.2 years (range: 22–43 years). Preoperative assessment of ovarian reserve revealed a mean Anti-Müllerian Hormone (AMH) level of 2.0 ± 2.3 ng/mL. A subset of patients presented with diminished ovarian reserve (AMH < 1 ng/mL), predominantly attributable to prior endometrioma surgery or extensive bilateral ovarian involvement. The burden of infertility in this cohort was substantial. Primary infertility was documented in 79.3% (*n* = 402) of cases, while 12.2% (*n* = 62) presented with secondary infertility. Notably, this cohort included a substantial proportion of women with recurrent or previously operated disease. Analysis of surgical history revealed that 23.7% (*n* = 118) of patients had undergone previous ovarian cystectomies for endometriomas, and 12.4% (*n* = 62) had prior surgeries specifically addressing pelvic pain. This high rate of re-intervention underscores the complexity of the surgical field, characterized by extensive fibrosis and distorted anatomy.

### 3.2. Surgical Phenotypes

All patients presented with histologically confirmed endometriosis. The disease severity was advanced, with a mean rASRM stage of 3.4 ± 0.9, indicating a predominance of Stage III and IV endometriosis. A distinguishing feature of this study is the high prevalence of posterior compartment involvement requiring colorectal dissection. As detailed in Table 1, nearly half of the cohort (47.5%, *n* = 241) required a bowel procedure to achieve complete macroscopic excision. Among the 241 women requiring bowel intervention, segmental colorectal resection was the most frequently performed procedure (*n* = 194, 38.3% of the total cohort). Rectal shaving was performed in 38 cases (7.5%), lateral rectal resection in 6 cases (1.2%), and extra-mucosal excision of bowel endometriosis (EMEB) in 3 cases (0.6%) (Table 1). In brief, segmental resection was performed for infiltrative lesions with deep muscular involvement and/or clinically significant luminal compromise; rectal shaving was used for superficial serosal/adventitial disease; and lateral rectal resection or EMEB were used in selected cases to achieve lesion excision while avoiding full-thickness bowel opening (as applicable).

### 3.3. Reproductive Outcomes

Overall Pregnancy and Miscarriage Rates: The primary endpoint, postoperative pregnancy, was evaluated in the final analysis cohort. During the follow-up period, 310 patients successfully achieved conception, corresponding to an overall pregnancy rate (PR) of 61.1%. Among the 310 clinical pregnancies, 12 (3.9%) resulted in spontaneous miscarriage. Live birth data were not available for the entire cohort at the time of analysis and are reported where documented.

**Figure 2:**
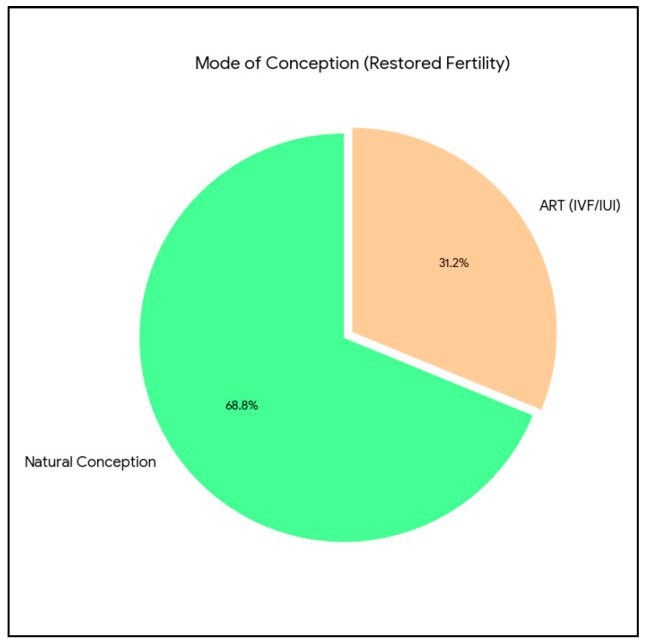
Mode of Conception.

Mode of Conception: A critical finding of this study is the predominant restoration of spontaneous fertility following surgery. Of the 310 women who conceived, 218 (70.3%) achieved pregnancy through natural conception, while 92 (29.7%) required assisted reproductive technologies, as we can see in Figure 2. Among the ART conceptions, the large majority were achieved through IVF/ICSI, with intrauterine insemination (IUI) accounting for a minority of cases.

Clinical pregnancy rates varied across bowel surgical strategies (Table 2). In women undergoing segmental colorectal resection (*n* = 194), 177 achieved a clinical pregnancy (91.2%). All 38 women treated with rectal shaving achieved a clinical pregnancy (100%). Among women managed with lateral rectal resection (*n* = 6), 6 achieved a clinical pregnancy (100%), whereas 1 of 3 women treated with extra-mucosal excision of bowel endometriosis (EMEB) achieved a clinical pregnancy (33.3%). Among women who did not require a bowel procedure (*n* = 266), 88 achieved a clinical pregnancy (33.1%). The overall difference in pregnancy rates between women undergoing any bowel procedure and those who did not was statistically significant (χ^2^ = 183.0, df = 1, *p* < 0.001; OR = 23.6, 95% CI [13.9–40.3]). Among women with bowel involvement, no statistically significant difference in pregnancy rate was observed between the segmental resection and rectal shaving subgroups (Fisher’s exact test, *p* = 0.083), supporting the premise that the extent of bowel resection does not adversely impact fertility outcomes when nerve-sparing principles are applied.

### 3.4. Factors Associated with Achieving Clinical Pregnancy

We performed a comparative analysis between patients who conceived (Pregnant Group, *n* = 310) and those who did not (Non-Pregnant Group, *n* = 197) to identify predictors of success (Table 3). Women who successfully conceived were significantly younger than those who did not (32.3 vs. 34.1 years; t = −3.80, *p* = 0.0002) and demonstrated significantly higher preoperative ovarian reserve (AMH 2.2 ± 2.3 vs. 1.4 ± 2.3 ng/mL; t = 3.82, *p* = 0.0002). The EFI score remained a robust independent predictor of conception, with a significantly higher mean score in the pregnant group (5.5 ± 1.9 vs. 4.9 ± 2.2; t = 3.15, *p* = 0.0017, mean difference 0.6, 95% CI [0.23–0.97]). Among women undergoing bowel surgery, the type of bowel procedure was not significantly associated with pregnancy outcome (Fisher’s exact test, *p* = 0.083), consistent with the hypothesis that nerve-sparing radicality preserves fertility irrespective of the extent of bowel resection.

## 4. Discussion

### 4.1. Principal Findings

The present study, conducted on a large cohort of 507 patients, demonstrates that minimally invasive excision of deep endometriosis (DE) is a highly effective fertility-sparing strategy. Our main finding is an overall pregnancy rate (PR) of 61.1%, with the majority of conceptions (70.3%) occurring spontaneously. These results challenge the growing tendency to bypass surgery in favor of primary Assisted Reproductive Technology (ART) for complex endometriosis. As argued by Taylor et al. [2] and Vercellini et al. [21], the clinical priority should be the restoration of natural fertility, especially since surgery addresses the underlying inflammatory process. Our data suggest that thorough restoration of pelvic anatomy, even when requiring radical bowel procedures, creates a biological environment favorable for natural conception, provided that ovarian reserve is preserved and nerve-sparing principles are rigorously applied [3,12].

### 4.2. The Safety of Radicality

One of the most debated topics in current literature is the impact of colorectal resection on fertility. Critics often argue that radical dissection may impair pelvic autonomic innervation and vascularization, potentially reducing ovarian function or uterine receptivity. However, our results firmly contradict this concern. In our cohort, the Segmental Resection group (*n* = 194) achieved a remarkable pregnancy rate of 91.2% among those with known outcomes. This is consistent with, and even exceeds, outcomes reported by other high-volume centers, such as the Rouen group, which reported pregnancy rates ranging from 46% to 60% after colorectal resection [11,17]. Long-term follow-up data, including those reported by Daraï et al. [15] and Bafort et al. [18], further support the durability of reproductive outcomes after laparoscopic bowel resection. We attribute these superior outcomes to two factors: 1—Systematic Nerve-Sparing Approach: The preservation of the hypogastric and pelvic splanchnic nerves is not incidental but a standardized step in our technique [3,16]. 2—Complete Disease Clearance: We hypothesize that leaving disease behind (incomplete shaving) maintains a pro-inflammatory peritoneal environment that is detrimental to gamete quality and implantation. This is supported by the molecular findings of Zou et al. [7], who identified immune cell dysregulation in the peritoneal fluid of DE patients, and Xiang et al. [9], who demonstrated that surgical excision favorably modulates endometrial gene expression and receptivity markers. By performing radical excision (segmental resection) when necessary, we effectively “reset” the pelvic environment.

### 4.3. Tailored Surgery

Our study highlights the shift towards a “tailored” surgical approach. Rather than applying a “one-size fits-all” technique, we utilized advanced procedures like Lateral Rectal Resection (stapled excision for eccentric nodules) and EMEB (mucosa-sparing excision). Although numbers are insufficient for statistical inference, these preliminary results suggest that advanced organ-sparing techniques may offer functional benefits without apparent compromise of fertility, warranting evaluation in larger prospective series. This approach aligns with the comparative analyses by Donnez and Roman [30] and de Paula Andres et al. [4], who emphasize that the choice of technique should be dictated by nodule size and infiltration depth rather than a fixed surgical dogma. This approach aligns with the recent expert consensus advocating for organ-sparing radicality [1,6], as well as comparative analyses emphasizing technique individualization over surgical dogma [4,30].

### 4.4. Natural Conception vs. ART

Perhaps the most impactful finding for patient counseling is the mode of conception. In our cohort, 70.3% of pregnancies were spontaneous. This is a critical argument in the cost-effectiveness debate. While upfront IVF is often promoted as a faster route to pregnancy, it carries significant financial and emotional burdens and does not address the underlying pain symptoms. Data from Maignien et al. [36] and Riemma et al. [20] suggest that while ART can achieve satisfactory birth rates, the cumulative success is often higher when the inflammatory burden of DE is surgically reduced. Our data support the strategy that surgery should be the first-line treatment for infertile women with DE and patent tubes, as it restores the potential for natural conception in more than two-thirds of cases. The EFI score proved to be a reliable tool in identifying these candidates, with a significantly higher mean EFI score in the pregnant group (5.5 ± 1.9 vs. 4.9 ± 2.2; *p* = 0.0017), consistent with the original validation by Adamson et al. [24] and subsequent meta-analyses by Vesali et al. [26] and Tomassetti et al. [25].

### 4.5. Strengths and Limitations

The primary strength of this study is the large sample size (*n* = 507) and the homogeneity of the surgical management, performed by a single high-volume multidisciplinary team. The exclusion of hysterectomies and the strict predefined eligibility criteria applied to the analysis cohort minimize selection bias. However, limitations exist. First, the retrospective design introduces inherent data collection biases. Second, while we report a robust pregnancy rate, the data regarding Live Birth Rates were not available for the entire cohort at the time of analysis. Third, we did not include a control group of patients treated with primary IVF without surgery, which limits direct comparison. Additionally, the variable duration of postoperative follow-up (range 12–84 months) precludes time-to-pregnancy analysis and may influence cumulative pregnancy rate estimates. The mode of conception was not documented in a subset of pregnant patients (*n* = 45, 14.5%), introducing potential misclassification bias in the spontaneous versus ART breakdown. Furthermore, the specific clinical indication prompting ART use (e.g., coexisting tubal factor, non-azoospermic male factor, diminished ovarian reserve, advanced maternal age, or patient preference for an accelerated route to pregnancy) was not consistently documented across all 92 women who conceived via assisted reproductive technologies, precluding a granular analysis of the factors driving this treatment pathway. Additionally, the cohort included a minority of patients (*n* = 43, 8.5%) without a formal in-fertility diagnosis who were included on the basis of an active desire for conception; given the small size of this subgroup, it was not feasible to perform a separately powered stratified analysis of pregnancy rate by infertility status, and these patients were therefore analyzed together with the broader fertility-sparing cohort. Future prospective studies with larger “desire-only” subgroups are warranted to formally compare reproductive outcomes between these populations. Finally, live birth rate data were incompletely captured, limiting the clinical interpretation of pregnancy outcomes. Nevertheless, the high rate of spontaneous conception serves as a strong internal validation of the surgical benefit.

## 5. Conclusions

Minimally invasive management of deep endometriosis (DE), when executed by a high-volume multidisciplinary team, represents a highly effective therapeutic strategy for the restoration of reproductive function. The results of this study demonstrate that comprehensive surgical excision enables a cumulative pregnancy rate of 61.1%, with the vast majority of conceptions occurring spontaneously. These data emphasize the capacity of surgical intervention to restore physiological pelvic balance, offering patients a viable and effective alternative to assisted reproductive technologies.

A fundamental contribution of this research is the invalidation of concerns regarding the negative impact of radical surgery on fertility. Segmental colorectal resection, integrated with rigorous nerve-sparing techniques, not only preserves reproductive potential but also facilitates success rates exceeding 90% in the analyzed cohort. Furthermore, the implementation of tailored, organ-sparing techniques—such as lateral rectal resection and extra-mucosal excision—marks a significant evolution in surgical precision, successfully combining complete disease eradication with optimal functional preservation.

In conclusion, comprehensive surgery should be considered the first-line therapeutic option for infertile patients with deep endometriosis. This approach offers a dual benefit: the definitive resolution of painful symptomatology and the restoration of natural fertility, thereby reducing dependency on assisted reproductive technologies. Additionally, the systematic utilization of the Endometriosis Fertility Index remains a fundamental pillar in postoperative management, providing essential prognostic benchmarks for personalized patient counseling and determining the optimal timing for intervention in their reproductive journey.

## Figures and Tables

**Table 1:** Baseline clinical and surgical characteristics of women undergoing fertility-sparing minimally invasive surgery for deep endometriosis (*n* = 507).

Characteristics	Values
Demographics	mean ± standard deviation
Age at Surgery (years), mean ± SD	33.0 ± 5.2
Body Mass Index (BMI), mean ± SD	23.4 ± 3.8
Ovarian Reserve	mean ± standard deviation
Preoperative AMH (ng/mL), mean ± SD	2.0 ± 2.3
Surgical History	Frequency (percentage)
Previous Cystectomy (Endometrioma), *n* (%)	118 (23.7%)
Previous Surgery for Infertility, *n* (%)	83 (16.7%)
Previous Surgery for Pelvic Pain, *n* (%)	62 (12.4%)
Infertility Type	Frequency (percentage)
Primary Infertility, *n* (%)	402 (79.3%)
Secondary Infertility, *n* (%)	62 (12.2%)
Disease Severity	
rASRM Stage, mean ± SD	3.4 ± 0.9
EFI score, mean ± SD	5.3 ± 2.0
Surgical Procedures	Frequency (percentage)
No Bowel Resection, *n* (%)	266 (52.5%)
Bowel Procedures (Total), *n* (%)	241 (47.5%)
- Segmental Resection	194 (38.3%)
- Rectal Shaving	38 (7.5%)
- Lateral Rectal Resection	6 (1.2%)
- EMEB (Mucosa-Sparing)	3 (0.6%)

Legend: Data are presented as mean ± standard deviation for continuous variables and number (percentage) for categorical variables. AMH = Anti-Müllerian Hormone; rASRM = Revised American Society for Reproductive Medicine stage; EMEB = Extra-Mucosal Excision of Bowel Endometriosis.

**Table 2:** Postoperative clinical pregnancy outcomes by bowel surgical strategy.

Surgical Strategy	Total *n*	Clinical Pregnancy *n* (%)	No Clinical Pregnancy *n* (%)
Overall cohort	507	310 (61.1)	197 (38.9)
No bowel procedure	266	88 (33.1)	178 (66.9)
Any bowel procedure	241	222 (92.1)	19 (7.9)
Segmental colorectal resection	194	177 (91.2)	17 (8.8)
Rectal shaving	38	38 (100.0)	0 (0.0)
Lateral rectal resection	6	6 (100.0)	0 (0.0)
EMEB (extra-mucosal excision)	3	1 (33.3)	2 (66.7)

**Table 3:** Comparison of key factors between women who achieved clinical pregnancy and those who did not. t = Student’s *t*-test statistic. * *p* < 0.001.

Variable	Pregnant (*n* = 310)	Non-Pregnant (*n* = 197)	Stat	*p* Value
Age at surgery (years)	32.3	34.1	t = −3.8	0.0002 *
Preoperative AMH (ng/mL)	2.2	1.4	t = 3.82	0.0002 *
EFI score (mean ± SD)	5.5 ± 1.9	4.9 ± 2.2	t = 3.15	0.0017

## Data Availability

The data are available from the corresponding author upon reasonable request.

## Abbreviations

The following abbreviations are used in this manuscript:

DE	Deep endometriosis
EFI	Endometriosis Fertility Index
PR	Pregnancy rate
ART	Assisted Reproductive Technology
Bcl-2	B-cell lymphoma 2
TVUS	Transvaginal ultrasound
MRI	Magnetic resonance imaging
EMEB	Extra-mucosal excision of bowel endometriosis
LARS	Low anterior resection syndrome
IVF	In vitro fertilization
ICSI	Intracytoplasmic sperm injection
AMH	Anti-Müllerian Hormone
